# Hall-Effect Based Semi-Fast AC On-Board Charging Equipment for Electric Vehicles

**DOI:** 10.3390/s111009313

**Published:** 2011-09-28

**Authors:** María Isabel Milanés-Montero, Javier Gallardo-Lozano, Enrique Romero-Cadaval, Eva González-Romera

**Affiliations:** Power Electrical and Electronic Systems Research Group, Escuela de Ingenierías Industriales, Universidad de Extremadura, Avda. de Elvas, s/n, Badajoz 06006, Spain; E-Mails: jagallardo@peandes.unex.es (J.G.-L.); eromero@unex.es (E.R.-C.); evagzlez@unex.es (E.G.-R.)

**Keywords:** V2G, G2V, electric vehicle, plug-in hybrid electric vehicle, battery charging equipment, power quality, perfect harmonic cancellation strategy

## Abstract

The expected increase in the penetration of electric vehicles (EV) and plug-in hybrid electric vehicles (PHEV) will produce unbalanced conditions, reactive power consumption and current harmonics drawn by the battery charging equipment, causing a great impact on the power quality of the future smart grid. A single-phase semi-fast electric vehicle battery charger is proposed in this paper. This ac on-board charging equipment can operate in grid-to-vehicle (G2V) mode, and also in vehicle-to-grid (V2G) mode, transferring the battery energy to the grid when the vehicle is parked. The charger is controlled with a Perfect Harmonic Cancellation (PHC) strategy, contributing to improve the grid power quality, since the current demanded or injected has no harmonic content and a high power factor. Hall-effect current and voltage transducers have been used in the sensor stage to carry out this control strategy. Experimental results with a laboratory prototype are presented.

## 1. Introduction

Alternative and sustainable transportation methods have become more attractive options in the last years due to the high fuel prices, environmental concerns and the demand for renewable energy. Widespread adoption of plug-in hybrid electric vehicles (PHEV) and electric vehicles (EV) could reduce greenhouse gas emissions from vehicles, decrease petroleum use and constitute an effective solution from the economical point of view, since the cost of electricity per mile of driving is less than one-fifth that of gasoline [1,2].

The development of battery charging equipment for these vehicles has been under research for the last decade [3–10]. However, most of the battery chargers behave as nonlinear loads, so the increase in the penetration of EV and PHEV will cause unbalanced conditions, reactive power consumption and current harmonics drawn by the battery chargers, producing disturbances on the electrical power system. These disturbances could cause line losses, heating problems, voltage stability problems, *etc*. [11]. The concerns about the impact of EV and PHEV on the future smart grid led the Electric Power Research Institute (EPRI) to publish the Recommended Practice TR-109023 [12], relating to charging equipment’s operational recommendations for power quality. This problem has also been assessed by the Society of Automotive Engineers (SAE) in the standard SAE J2894 [13], which is scheduled to be completed in 2011. This standard collects the power quality and charger efficiency requirements based on EPRI legacy recommendation. Compared to the EPRI document, the SAE requirements are more exigent, since the power transfer efficiency of the charging equipment has been increased from 85% to 90% and the Total Harmonic Distortion (THD) of the charger has been reduced from 20% to 10%, maintaining the power factor in the 95%. In order to comply with this standard, it is necessary to develop new battery charging equipment with higher power quality. In this way, novel control strategies capable to meet the requirements of the standard have to be implemented in the coming years. These chargers will reduce the impact of EVs and PHEVs on the electric infrastructure, eliminating the non-linear behavior of actual battery chargers and achieving higher power transfer efficiency, minimizing line losses in the power system.

A bidirectional battery charger has two main functions: charging the battery to a proper state of charge (SOC). This operation mode is called charging mode or grid-to-vehicle (G2V) mode. The other operation is called discharging mode or vehicle-to-grid (V2G) mode, which means the battery energy can be inverted and flows back to the grid. Additional functions have been proposed in the literature, such as vehicle-to-home (V2H) mode, supplying ac electricity to a house for a short time in case of grid failure [14] or active filtering capability, reducing harmonic and reactive power consumption at the point of coupling, contributing to improve the power quality locally [15,16].

In this paper, a high efficiency and high power factor bidirectional charging equipment is proposed with a simple and low-cost topology and provided with a novel control strategy to reduce the harmonic current demanded or injected into the grid. The objectives of the charger are to meet the present standards [12,13] and the requirements for charging of EV and PHEV in case of semi-fast devices, which are [17]: type: AC, power: 3.7–7.4 kW, duration: 7 h/100 km, voltage: 230 V and current 16–32 A.

The paper is organized as follows: a description of the power stage, control stage and sensor stage is provided in Section 2. The control stage comprises a novel control strategy to comply with the proposed specifications and the sensor stage includes the current and voltage transducers to carry out the control algorithms. Section 3 describes the experimental laboratory prototype used to validate the operation of the charging equipment. Experimental results under different voltage conditions are shown in Section 4. Finally, our conclusions are summarized in Section 5.

## 2. Charging Equipment Architecture

In this section, the on-board bidirectional charging equipment architecture is described. It is formed by three parts: the power stage, the control stage and the sensor stage.

### 2.1. Power Stage

**Figure 1.**
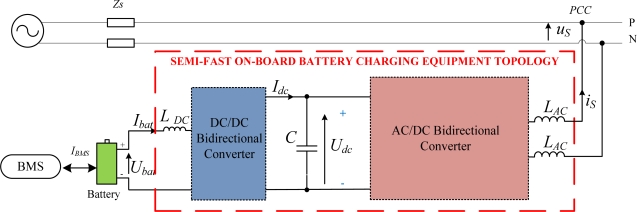
Single-phase battery charger topology.

The topology of the bidirectional single-phase on board battery charger is shown in Figure 1. It is formed by a bidirectional DC/DC converter and a bidirectional AC/DC converter, sharing the DC bus. The AC/DC converter is a full-bridge inverter, while de DC/DC converter is a half-bridge one. Ripple filters are placed in the DC and AC parts of the charger, to filter out the high switching frequency of the converters. These filters are modeled in the scheme of Figure 1 as an inductance (*L_DC_* or *L_AC_*) in series with the internal resistance (*R_DC_* or *R_AC_*). The advantages of this hard switched charger topology are the simplicity and cost, since it has fewer components (only 6 IGBTs and 6 diodes) compared to more complex topologies [18]. Besides, although there are other topologies proposed in literature with higher power efficiency, this one has a transfer power ratio enough to meet the SAE standard [16].

The battery receives the reference current, *I_BMS_*, from the Battery Management System (BMS). The sign criterium considered for the battery charger is: *I_bat_* < 0 in G2V mode, while in V2G mode, *I_bat_* > 0. In the G2V operation mode, the charging equipment has to demand power from the grid to charge the battery. In the 2GV operation mode, the excess of power stored in the battery that has not been used by the electric vehicle can be injected into the grid. It is expected to be a high efficiency charger, which means that power losses should be minimized as much as possible. It will be a target to attain by the control strategies of both converters.

### 2.2. Control Stage

The specifications to be achieved by the charger control stage are: current demanded or injected into the low voltage grid sinusoidal, in phase with the fundamental point of common coupling (PCC) voltage, with the aim of attaining:

- Unity displacement power factor (*PF*_1_ = 1);
- Charger current harmonic distortion low (*THD*_i_ < 5%);
- High efficiency ratio (*η* > 90%).

The control stage is composed by the control strategies, which generates the reference currents, and the current controllers, responsible for obtaining the switching signals of the converters, based on the comparison of the reference and measured currents.

No control strategy is necessary for the DC/DC converter, since the reference current for this equipment is obtained from the BMS, so *I_bat,ref_* = *I_BMS_*. The control strategy for the AC/DC converter is a Perfect Harmonic Cancellation (PHC) [19,20] control algorithm. This strategy assures that the current demanded or injected into the grid by the charging equipment will have no harmonic content and unity displacement power factor, meeting the specifications proposed for the charger.

The power in the DC side of the charging equipment, *P_DC_*, equals the power of the battery, *P_bat_*:

(1) $$P_{\mathrm{DC}}=P_{\mathrm{bat}}=U_{\mathrm{bat}}I_{\mathrm{bat}}$$

On the other hand, the instantaneous power in the AC side of the charger, *p_AC_*, can be calculated as the product of the instantaneous phase-to-neutral source voltage by the instantaneous source current, as:

(2) $$p_{\mathrm{AC}}=u_{S}i_{S}$$

Taking into account the charging equipment specifications, the source current will be sinusoidal and in phase with the fundamental source voltage, so the active or constant power in the AC side of the charger will be:

(3) $$P_{\mathrm{AC}}=U_{S1}I_{S}$$

where *U*_*S*1_ is the RMS value of the fundamental source voltage and *I_S_* the RMS value of the source current.

Neglecting power losses in the converters, conductors and filter inductors, the constant power in the DC side of the charging equipment (output of the battery) should be equal to the active power in the AC side of the charger (grid side), so from Equations (1) and (3), it gives:

(4) $$U_{\mathrm{bat}}I_{\mathrm{bat}}=U_{S1}I_{S}$$

Applying the PHC control strategy, the reference source current for the AC/DC converter, *i_S,ref_*, has to be in phase with the fundamental phase-to-neutral source voltage, so the instantaneous value is calculated multiplying the RMS value by a unity vector of the direction of the fundamental source voltage:

(5) $$i_{S,\mathrm{ref}}=I_{S}{\bar{u}}_{S1}$$

Obtaining the unity vector dividing the instantaneous value by the modulus of the vector (the RMS value):

(6) $${\bar{u}}_{S1}=\frac{u_{S1}}{U_{S1}}$$

and applying Equations (4) and (6) to (5), the reference source current is obtained as:

(7) $$i_{S,\mathrm{ref}}=\frac{U_{\mathrm{bat}}I_{\mathrm{bat}}}{U_{S1}^{2}}u_{S1}$$

To guarantee the proper operation of the AC/DC converter, the DC bus voltage has to maintain a constant value and satisfy a condition which depends on the converter topology and the grid voltage (in case of a full-bridge inverter, *U_DC_* has to be higher than the peak value of the source voltage). A small amount of current has to be absorbed from the grid in order to ascertain that the DC bus voltage maintains its reference value, *U_DC,ref_*. An additional block to control the DC bus voltage has been added in the control strategy. It is formed by a Proportional-Integral (PI) controller whose input is the error between the measured DC bus voltage and its reference value. In stationary state, when the error is zero, the output of the converter has a constant value, *K_C_*. The transfer function of this PI controller is *K_p_* + *K_i_*/*s*, being *K_p_* and *K_i_* the proportional and integral constants, respectively, of the controller. Since a sinusoidal current in phase with the source voltage should be demanded from the grid, the additional reference source current necessary to control the DC bus voltage, Δ*i_S,ref_*, is calculated multiplying the output of the PI controller by the instantaneous source voltage, resulting in:

(8) $${\Delta i}_{S,\mathrm{ref}}=K_{C}u_{S1}$$

Finally, from Equations (7) and (8), the proposed reference source current for the AC/DC converter is:

(9) $$i_{S,\mathrm{ref}}=\left(\frac{U_{\mathrm{bat}}I_{\mathrm{bat}}}{U_{S1}^{2}}+K_{C}\right)u_{S1}$$

**Figure 2.**
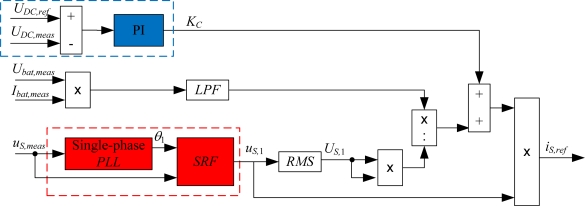
AC/DC converter control strategy.

The control scheme of the AC/DC converter strategy is shown in Figure 2. The blocks used to control the DC bus voltage are highlighted in blue. To extract the fundamental component of the measured source voltage, a single-phase Phase Locked Loop (PLL) system and a Synchronous Reference Frame (SRF) have been employed. The output of the single-phase PLL is the phase angle of the input fundamental component, *θ*_1_. Once this phase angle is known, the SRF is able to obtain the fundamental component of an input variable [21,22]. These blocks are highlighted in red in Figure 2. Finally, since the battery current has a ripple value due to the switching frequency of the DC/DC converter, a second order Butterworth Low-Pass-Filter (LPF) has been employed in the control strategy (see Figure 2) to filter out the ripple that appears in the power of the DC side of the charging equipment.

Fixed band, variable frequency hysteresis current controllers have been used in the DC/DC converter and AC/DC converter. In these controllers, the measured converter current is compared to a reference value. Depending on the sign of this comparison, the switching signals are generated.

**Figure 3.**
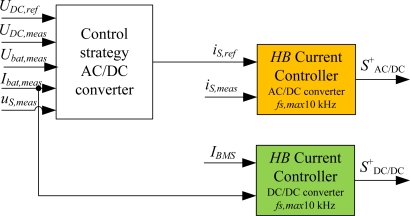
Control stage: control strategies and current controllers.

In case of the DC/DC converter the reference current is the current proposed by the BMS, *I_BMS_*, which is compared to the measured battery current, *I_bat_*. This controller is highlighted in green in Figure 3. The output of this block is the switching signal, *S_DC/DC_*^+^, which controls the two switches of the DC/DC converter.

**Figure 4.**
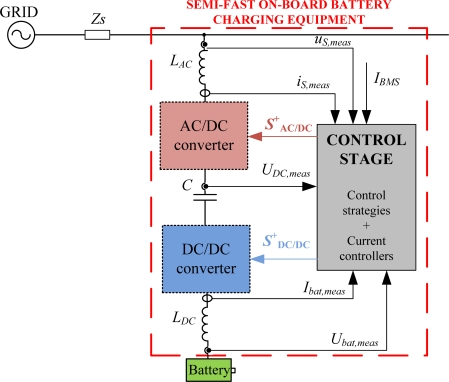
Semi-fast on-board battery charging equipment.

On the other hand, in case of the AC/DC converter, the reference current, *i_Sref_*, is obtained in the control strategy, and compared to the measured source current, *i_S_*. This controller is highlighted in yellow in the block diagram of the control stage displayed in Figure 3. The AC/DC converter is a full-bridge inverter controlled with only one switching signal, *S_AC/DC_*^+^. The global scheme of the semi-fast on-board battery charging equipment is shown in Figure 4. This scheme collects the power stage in addition to the control stage.

### 2.3. Sensor Stage

In the charging equipment, the sensor stage is composed by the current and voltage transducers and auxiliary elements necessary to supply the sensors. Hall-effect transducers are typically used in electric power applications [23,24], since they are immune to dust, dirt, mud and water, constituting a better option compared to optical and electromechanical sensing devices. The main advantage when using the Hall effect in current transducers is that it is possible to create a non-contacting current sensor. The transducer provides a voltage proportional to the current being sensed, without the additional resistance in the primary circuit required for the most common current sensing method. Besides, the voltage on the line to be sensed is not transmitted to the sensor, which enhances the safety of the measuring equipment.

Hall-effect sensors require signal conditioning to make the output usable for most applications. The signal conditioning electronics needed are a differential amplifier with low noise, high input impedance and moderate gain and temperature compensation.

From the previous section, it is easy to extract the variables which need to be measured for the control strategy. These variables are the battery current, *I_bat_*, the source current, *i_S_*, the battery voltage, *U_bat_*, the source voltage, *u_S_* and the DC bus voltage, *U_DC_*, so two current sensors and three voltage sensors has to be employed in the sensor stage. The location of these sensing devices required for the charging equipment is displayed in Figure 4.

## 3. Charging Equipment Laboratory Prototype

**Figure 5.**
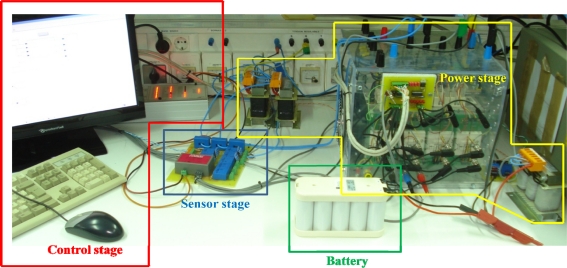
Laboratory prototype of the semi-fast charging equipment.

An experimental laboratory prototype to validate the performance of the semi-fast charging equipment has been built (see Figure 5). The electrical parameters of this bidirectional charger prototype are shown in Figure 1 and summarized in Table 1. From this table, one can notice that it is a scaled-down 1:10 prototype to make low power tests in the laboratory. The parameters of the battery pack used in the experimental setup are collected in Table 2 and highlighted in green in Figure 5. It is a nickel-metal hydride (Ni-MH) battery formed by 10 VHT F (SAFT) cells.

A four-leg SEMIKRON inverter has been used in the power stage. Only three of these legs are employed for the DC/DC and AC/DC converters. Ferromagnetic core inductors are used as filter inductors. The power stage of the laboratory prototype is highlighted in yellow in Figure 5. DS1104 (dSPACE) has been employed as the real time control platform in the control stage. This control platform is highlighted in red in Figure 5.

**Figure 6.**
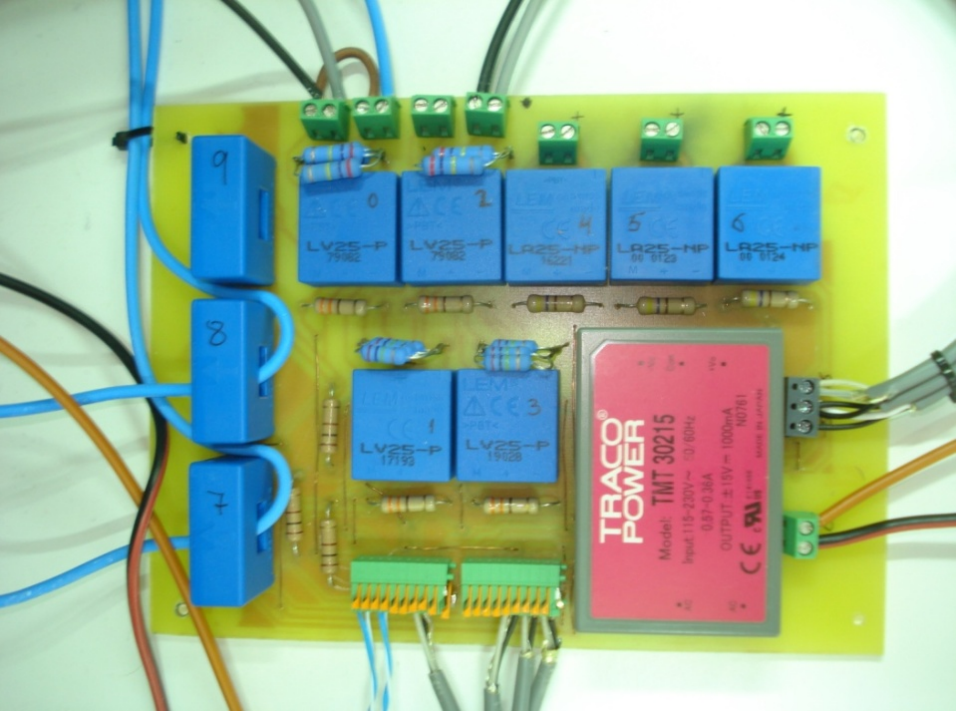
Sensor card used in the experimental setup to measure the control variables.

The sensor card is highlighted in blue in Figure 5 and displayed in Figure 6. It is a general-purpose card with six current transducers and four voltage transducers. For the experimental setup it is necessary to measure two currents (one DC current and one AC current) and three voltages (two DC voltages and one AC voltage). Hall-effect current transducers LA 55-P (LEM) for the electronic measurement of the DC current *I_bat_* and the AC current *i_S_*, with galvanic isolation between the high power circuit and the electronic circuit, have been used. These sensors operate with very good linearity, no losses and high immunity to external interferences. The electrical parameters of this current transducer are summarized in Table 3.

To measure the DC voltages *U_bat_* and *U_DC_* and the AC voltage *u_S_*, Hall-effect voltage transducers LV 25-P (LEM) have been employed. This transducer has galvanic isolation between the high voltage circuit and the electronic circuit and works with excellent accuracy, very good linearity and low disturbance in common mode. Table 3 collects the electrical data of this voltage sensor.

## 4. Results and Discussion

Experimental tests under ideal and distorted source voltage have been conducted to validate the battery charging equipment operation using the small-scale laboratory prototype. In the following sections the experiments are explained and the results discussed.

### 4.1. Sinusoidal Source Voltage

**Figure 7.**
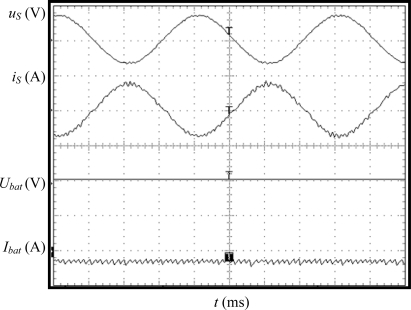
Experimental results in case of sinusoidal source voltage in G2V mode. Y-axis (from top to bottom): grid voltage (50 V/div), charging equipment current (5 A/div), battery voltage (10 V/div), battery current (20 A/div). X-axis: time (5 ms/div).

Firstly the charging equipment prototype has been tested under ideal source voltage, without harmonic distortion. Experimental results in case of G2V mode are displayed in Figure 7. In this figure are shown, from top to bottom, the grid phase-to-neutral voltage, charger AC current, battery voltage and battery current. It can be observed that the charger current is almost sinusoidal and in contra-phase with the source voltage, which in this case has only fundamental component. The battery current is negative (−5 A), taking into account the proposed sign criterion. The Total Harmonic Distortion (THD) of the source current is 3.1%, the displacement power factor is 0.99 and the efficiency ratio is 91%, meeting the specifications proposed for the charging equipment.

**Figure 8.**
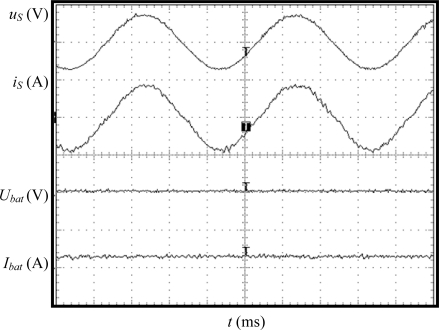
Experimental results in case of sinusoidal source voltage in V2G mode. Y-axis (from top to bottom): grid voltage (50 V/div), charging equipment current (5 A/div), battery voltage (10 V/div), battery current (20 A/div). X-axis: time (5 ms/div).

The results obtained when testing the V2G mode in case of ideal source voltage are shown in Figure 8. In this case the charger current is in phase with the source voltage and without harmonics. The battery current has a positive value of 5 A. The THD of the charger current is 3.2%, the displacement power factor is unity and the efficiency ratio is 92%.

### 4.2. Distorted Source Voltage

The main contribution of the charging equipment proposed in this paper is the capability of the charger to behave as a linear load/source in case of distorted source voltage, demanding/injecting a current without harmonic distortion. In order to test experimentally this ability, the charger has been proved under distorted source voltage. A programmable source (HP 6834B, 4.5 kVA) has been employed to generate a single-phase voltage with the desired harmonic content. This equipment does not allow injecting current into the grid, so only the G2V operation mode has been carried out. The source voltage has been programmed with 20% 5th and 10% 11th harmonic components, so the THD of the source voltage has been fixed in 22.36%.

**Figure 9.**
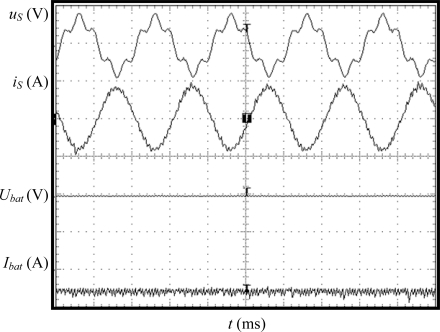
Experimental results in case of distorted source voltage in G2V mode. Y-axis (from top to bottom): grid voltage (50 V/div), charging equipment current (5 A/div), battery voltage (10 V/div), battery current (10 A/div). X-axis: time (10 ms/div).

Experimental results are displayed in Figure 9. The charger current is sinusoidal and in contra-phase with the fundamental source voltage. The battery current is negative (−5 A), since the charging mode is tested. The THD of the charger current is 3.4%, the displacement power factor is unity and the efficiency ratio is 90%. Although the experimental conditions are very exigent, since the source voltage distortion is very high, the charger demands from the grid a current with high quality and minimum losses.

## 5. Conclusions

A novel control strategy for an on-board semi-fast battery charging equipment has been proposed in this paper. This strategy allows the charger to operate with G2V and V2G modes, behaving as a linear load or linear source, since the charger current is sinusoidal, regardless the quality of the voltage at the point of coupling. The control strategy has been implemented for a simple single-phase topology, requiring a low number of measurements in the control stage. The proposed power and control stages have been described. Hall-effect current and voltage transducers have been used in the sensor stage. Finally, the topology and control have been tested in a laboratory prototype, charging and discharging a Ni-MH battery pack. The performance of the charger has been validated empirically under ideal and distorted source voltage conditions, with good results.

At present we are working to test the charger prototype in 1:1 scale experiments, and use it in a commercial car. The battery manufacturer is designing for our tests a battery module formed by 25 10 VHT F packs connected in series, so *U_bat_* = 300 V. In this case, the parameters of the charger will be 230 V, 16 A, 3.7 kW, complying with the requirements of semi-fast devices. It is expected that the results in this high power tests will be the same as the ones obtained in the low power laboratory experiments.

## Figures and Tables

**Table 1.** Electrical parameters of the charging equipment.

**AC output**		**DC input**	

Nominal source voltage (RMS):	23 V	Nominal battery pack voltage:	12 V
Nominal source current (RMS):	2.6 A	Charge/Discharge current:	−5 A/5 A
Nominal output power:	60 W	Nominal input power:	60 W
*L_AC_*	15.85 mH	*L_DC_*	10.54 mH
*R_AC_*	0.38 Ω	*R_DC_*	0.15 Ω

**Table 2.** Electrical parameters of the Ni-MH battery pack.

Manufacturer:	SAFT	Maximum voltage (charge mode):	14.5 V
Battery Pack:	10 VHTF	Maximum charge current:	5 A
Nominal voltage:	12 V	Minimum voltage (charge mode):	8 V
Nominal capacity:	10 Ah	Maximum discharge current:	40 A

**Table 3.** Electrical parameters of the current and voltage transducers.

**Current transducers**		**Voltage transducers**	

Primary nominal current (RMS):	50 A	Primary nominal current (RMS):	10 mA
Measurement range:	0 ... ±70 A	Measurement range:	0 ... 500 V
Conversion ratio:	191,4 A/V	Conversion ratio:	1000 V/V
